# The prevalence of common mental disorders and PTSD in the UK military: using data from a clinical interview-based study

**DOI:** 10.1186/1471-244X-9-68

**Published:** 2009-10-30

**Authors:** Amy C Iversen, Lauren van Staden, Jamie Hacker Hughes, Tess Browne, Lisa Hull, John Hall, Neil Greenberg, Roberto J Rona, Matthew Hotopf, Simon Wessely, Nicola T Fear

**Affiliations:** 1 King's Centre for Military Health Research, Institute of Psychiatry, Department of Psychological Medicine, Cutcombe Road, Denmark Hill, London, SE5 9RJ, UK; 2 Academic Centre for Defence Mental Health, Institute of Psychiatry, Department of Psychological Medicine, Cutcombe Road, Denmark Hill, London, SE5 9RJ, UK; 3 Health *Care *and Social Care Advisory Service (HASCAS), 11-13 Cavendish Square, London W1G 0AN, UK

## Abstract

**Background:**

The mental health of the Armed Forces is an important issue of both academic and public interest. The aims of this study are to: a) assess the prevalence and risk factors for common mental disorders and post traumatic stress disorder (PTSD) symptoms, during the main fighting period of the Iraq War (TELIC 1) and later deployments to Iraq or elsewhere and enlistment status (regular or reserve), and b) compare the prevalence of depression, PTSD symptoms and suicidal ideation in regular and reserve UK Army personnel who deployed to Iraq with their US counterparts.

**Methods:**

Participants were drawn from a large UK military health study using a standard two phase survey technique stratified by deployment status and engagement type. Participants undertook a structured telephone interview including the Patient Health Questionnaire (PHQ) and a short measure of PTSD (Primary Care PTSD, PC-PTSD). The response rate was 76% (821 participants).

**Results:**

The weighted prevalence of common mental disorders and PTSD symptoms was 27.2% and 4.8%, respectively. The most common diagnoses were alcohol abuse (18.0%) and neurotic disorders (13.5%). There was no health effect of deploying for regular personnel, but an increased risk of PTSD for reservists who deployed to Iraq and other recent deployments compared to reservists who did not deploy. The prevalence of depression, PTSD symptoms and subjective poor health were similar between regular US and UK Iraq combatants.

**Conclusion:**

The most common mental disorders in the UK military are alcohol abuse and neurotic disorders. The prevalence of PTSD symptoms remains low in the UK military, but reservists are at greater risk of psychiatric injury than regular personnel.

## Background

The mental health of any fighting force influences their occupational effectiveness. It has been shown to be an essential factor in the retention and productivity of military personnel [1] and increases the chance of social exclusion for those who leave the Armed Forces [2,3].

Many factors, including deployment and combat, are known to increase the risk of psychological distress and psychiatric injury [4,5]. Recent US reports indicate that the prevalence of mental disorders after deployment to Iraq and Afghanistan is particularly high and rising [4,6].

Since the beginning of the Iraq conflict, over 100,000 UK reserve and regular Service personnel have been deployed to Iraq and Afghanistan. It is likely that these personnel are at increased risk of operational stress injury but detailed clinical data about the specific heath needs of those who have deployed is lacking in the UK. These data are important for health service planners, providers and policy makers. Routinely collected data based on presentation to health care providers is problematic, since many are reluctant to disclose mental disorders within the military environment [7,8].

We have previously reported that deployment to the Iraq War was not associated with poorer health outcomes for regular personnel, but there is evidence of an effect on the health of reservists [9]. However, the study was based on symptoms obtained by self-report questionnaire rather than interview-based measures and did not examine the prevalence of specific mental disorders. Thus, there is a need to confirm these results by ascertaining the prevalence of psychiatric disorders in a large epidemiological study using detailed standardized diagnostic assessments.

The aim of this study was to assess the prevalence of specific common mental disorders and post traumatic stress disorder (PTSD) symptoms and associated risk factors in UK Service personnel using a two-stage epidemiological sampling technique [9]. A second aim of our study was to compare the prevalence of depression, PTSD symptoms, health perception and suicidal ideation in regular and reserve UK Army personnel who deployed to Iraq with their US counterparts.

## Methods

### Study Population

This study was based on a sample drawn from Phase 1 of the King's College Military Health Research (KCMHR) Military Health study. Full details can be found in Hotopf et al [9]. In brief, the study was the first phase of a cohort study of UK military personnel in service at the time of the 2003 Iraq War (Operation TELIC, the military codename for the current operation in Iraq). In total, 4722 regular and reserve personnel who were deployed on TELIC 1 (the war-fighting phase) and 5550 regular and reserve personnel who were not deployed on TELIC 1 completed a questionnaire between June 2004 and March 2006 on their military and deployment experiences, lifestyle factors and health outcomes. TELIC 1 was defined, for the purposes of this study, as the period 18^th ^January 2003 to 28^th ^April 2003. A proportion of the study participants were subsequently deployed (i.e. TELIC 2-6) whose mission was counter-insurgency rather than war fighting. The response rate for the Phase 1 study was 61%.

**Figure 1:**
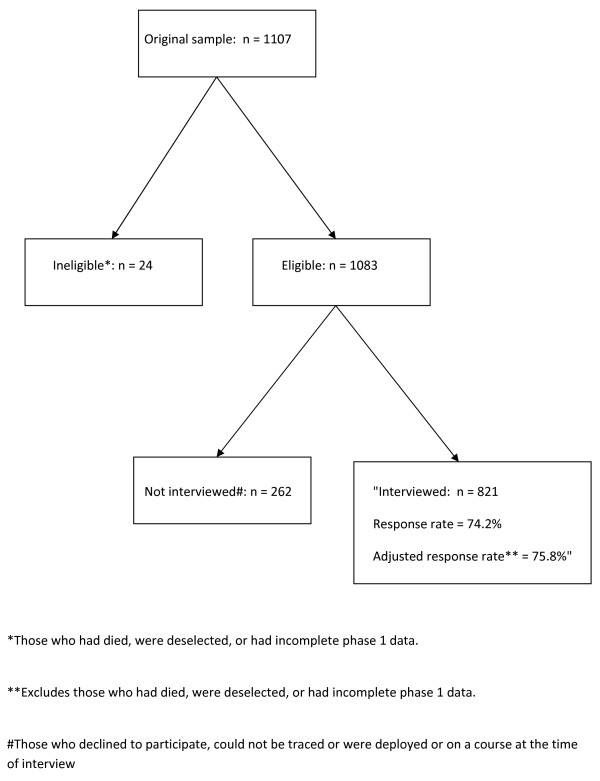
**Flow of participants from the original nested sample to the final sample**.

The participants for the current study were drawn from those who completed questionnaires from the phase 1 of the KCMHR military health study and consented to follow up. We used a 'two-phase survey' technique [10] to identify the prevalence of psychiatric diagnoses in the whole KCMHR military health study sample. Possible psychiatric cases were identified from the main cohort using the 12-item General Health Questionnaire (GHQ) [11]. A random sample of those who scored above the threshold for 'GHQ caseness' (score ≥ 3) were selected for interview, together with a random sample of the non-GHQ cases. Cases were over-sampled; 70% of the final sample for the study were GHQ cases, and 30% were non-GHQ cases. We also included all participants who scored ≥ 50 [12] on the Post Traumatic Stress Disorder Checklist (PCL). There are a variety of different cut-offs used for the PCL, but a cut-off of 50 has been widely used in both UK [9] and US military studies [4]. To ensure adequate power to make statistical inferences, we stratified the sample by regular/reserve status (50% each), and deployment status (50% deployed on TELIC 1, 50% deployed elsewhere or were not deployed). In all other respects, group participants were representative of the KCMHR military health study responders with regards to Service branch and demographic characteristics (age, rank, ethnicity) and in turn the main study was representative of the UK military in 2003 [9]. The final sample size was 821 (see Figure 1).

### Participant Tracing

Participants were approached through an invitation pack that was sent either to their civilian address for ex-Service and reservist personnel or the most recent military addresses for still serving personnel. Numerous methods were used to facilitate response (van Staden L, Iversen A, Fear NT, Hall JW, Wessely S: "50 Ways trace your veteran"; increasing participation in research can be cheap and effective, submitted). Participants were offered a cheque or supermarket voucher for £15 to compensate them for their time.

### Survey instrument

A telephone interview schedule was designed to be completed within 45 minutes. The interview schedule included deployment experience since 2003, the Patient Health Questionnaire (PHQ) [13] and an additional measure for the diagnosis of PTSD (the 4-item Primary Care PTSD or PC-PTSD) [14].

The PHQ[13] is a structured diagnostic instrument which can either be self-administered on paper or by computer or can be completed on the participant's behalf by a lay-researcher. It is validated for telephone use [15] and can be used to give both continuous scores for depression (PHQ 9 Depression severity score), and somatisation (somatic symptom severity score) and categorical scores for the presence of a major depressive illness, other depressive illness, panic disorder, generalised anxiety disorder, somatoform disorder, and alcohol 'abuse'. It has been used previously in both UK [16] and US military samples [4].

For PTSD symptoms, we used a 4-item measure developed for primary care by the National Center for PTSD (Primary Care PTSD Screen, PC-PTSD) [17]. The screen has been used in the Post Deployment Health Assessment (PDHA) [6] and Post Deployment Reassessment (PDHRA) mandated by the US Department of Defense [18]. In this study, we chose a cut-off of three or more to define PTSD symptoms as a recent study has demonstrated that this cut-off provides a high specificity (0.88) with acceptable sensitivity (0.76) [19]. This cut-off has recently been used by a US study looking at troops who have recently returned home from operations [20]. We included a lifetime DSM-IV Criterion A1 event screening question taken from the National Comorbidity Study [21]. If participants did not endorse a Criterion A1 event during their lifetime, they were not invited to complete the PC-PTSD. 61.9% (weighted prevalence) of the KCMHR military health study reported any Criterion A1 event. All interviews were conducted during 2006 and 2007.

### Sample size

We estimated that with a sample size of 626, the study would have adequate power (80%) to detect a difference of 10 percentage points (with an alpha of 0.05) in the prevalence of psychiatric disorders between those who deployed to the main fighting period of the Iraq War and those who did not (assuming a prevalence of common mental disorders of 20%). This allowed for a 60% response rate. The final study had greater statistical power than envisaged as the final response rate was 76%.

### Analysis

All statistical analyses were undertaken using the statistical software package STATA (version 10.0) [22]. Weighted and unweighted (not shown) prevalence estimates of mental disorders were calculated for the phase 1 KCMHR military health study. Weighting was based on the inverse of the sampling weight for the three characteristics that were over-sampled in the study compared to the cohort sample (reserve status, GHQ caseness, PCL caseness). Details of the sampling weights used are shown in Additional file 1: table S1.

Information on educational attainment, childhood adversity, Service branch, role in parent unit and engagement type were obtained at phase 1 (via the self-completion questionnaires). The childhood adversity measure included a series of questions about experiences in childhood. These have been described in depth in another paper [23] but consist of 16 questions with the stem: "when I was growing up...". Participants were given a choice of answering true or false to each item. Care was taken to include both protective and adverse experiences in childhood, with examples including: coming from a close family, playing truant from school, or being hit by parents or caregivers regularly. For the purposes of this study we use a composite score of adverse childhood events with higher scores indicating greater adversity (items were reverse scored as appropriate).

Weighted prevalence, adjusted odds ratios with their 95% confidence intervals are presented for the defined diagnostic categories. Uni-variable analyses were initially undertaken to examine associations between the diagnoses of common mental disorders and PTSD. Multi-variable logistic regression was then used to control for confounders for psychiatric disorder. All analyses were undertaken using the svy command in STATA to take account of sampling weights (as shown in Additional file 1: table S1).

### Ethical Issues

The study received approval from both the King's College Hospital NHS Research Ethics Committee (ref: 05/Q0703/155) and also from the Ministry of Defence (Navy) Personnel Research Ethics Committee (ref: 0522/22).

## Results

### Response rate (Figure 1)

Twenty four of the 1107 participants were ineligible (10 were deselected due to invalid selection into the Phase 1 study, 3 had died and 11 had incomplete Phase 1 data for their PCL or GHQ responses (Figure 1)). One hundred and eleven declined to participate, 127 could not be traced despite multiple attempts and 24 were unavailable during the interview period due to deployment/training. The final sample consisted of 821 participants. The response rate was 74.2% and the adjusted rate was 75.8%.

### The characteristics of study responders (Additional file 1: table S2)

Uni-variable analysis of responders and non-responders based on their phase 1 questionnaire responses showed that compared to responders, non-responders were younger, more likely to hold a lower rank, and be regular personnel. After adjustment, only younger age remained a significant predictor of non-response. There was no difference in phase 1 health outcomes between those who responded and those who did not.

### The prevalence of common mental disorders and PTSD symptoms (Table 1)

**Table 1:** Mental health disorders in the KCMHR military health study, weighted^a ^prevalence (%) and 95% confidence interval (CI)

**Diagnosis**	**Prevalence, %**	**95% CI**
Any PHQ diagnosis or PTSD	28.9	24.6-33.7

PTSD symptoms	4.8	3.3-7.1

Any PHQ diagnosis	27.2	23.0-31.9

Any neurotic disorder^d^	13.5	10.6-17.1

Any depressive syndrome^b^	11.0	8.2-14.5
Major depressive syndrome	3.7	2.4-5.8
Other depressive syndrome	7.3	5.0-10.4

Any anxiety syndrome^c^	4.5	3.1-6.5
Panic syndrome	1.1	0.6-2.2
Other anxiety syndrome	3.8	2.5-5.7

Somatisation disorder	1.8	1.0-3.4

Alcohol abuse	18.0	14.5-22.3

^a ^Weighted to take account of sample weights

^b ^Major depressive syndrome or other depressive syndrome

^c ^Panic syndrome or other anxiety syndrome

^d ^Any depressive syndrome, any anxiety syndrome or somatisation disorder

The weighted prevalence of any common mental disorder or PTSD symptoms in the phase 1 KCMHR military health study was 28.9%, and 4.8% for PTSD symptoms. Alcohol abuse was the most common mental disorder (18.0%), followed by any neurotic disorder (13.5%). Within neurotic disorders, major depressive disorder was less common than milder depressive disorders (3.7% versus 7.3%). Panic (1.1%) and somatisation di sorders (1.8%) were relatively uncommon.

### Socio-demographic and military correlates of mental disorders (Tables 2 and 3)

**Table 2:** Weighted^a ^prevalence of mental health disorders in the KCMHR military health study by key demographics, % and 95% confidence intervals (CI)

**Variable**	**PTSD symptoms**		**Any neurotic disorder^d^**		**Alcohol abuse**	
	**%**	**95% CI**	**%**	**95% CI**	**%**	**95% CI**
Age at interview (years)						
< 30	5.4%	1.9 - 14.2	23.2%	13.9 - 36.3	44.1%	31.2 - 57.8
30-34	8.8%	3.6 - 20.1	11.1%	5.6 - 20.6	19.3%	11.0 - 31.6
35-39	2.7%	1.6 - 4.5	14.7%	8.6 - 24.2	18.4%	11.3 - 28.7
40-44	5.5%	2.9 - 10.3	12.6%	7.5 - 20.4	12.0%	7.0 - 20.0
45+	2.9%	1.3 - 6.3	9.3%	5.3 - 16.0	7.2%	3.8 - 13.2
p-value^b^	0.18		0.13		< 0.01	
						
Sex						
Male	5.1%	3.4 - 7.7	14.1%	10.9 - 18.1	19.5%	15.5 - 24.1
Female	1.9%	0.8 - 4.2	8.4%	4.1 - 16.3	5.9%	2.3 - 14.0
p-value^b^	0.02		0.15		< 0.01	
						
Educational attainment at phase 1						
No qualifications	18.4%	7.3 - 39.1	23.3%	11.9 - 40.6	28.4%	13.7 - 49.7
O level equivalent	4.3%	2.3 - 8.0	15.0%	10.2 - 21.7	23.2%	16.6 - 31.3
A level equivalent	2.8%	1.7 - 4.5	15.7%	9.6 - 24.6	14.9%	9.2 - 23.4
Degree	5.2%	2.3 - 11.3	6.9%	3.9 - 12.1	13.1%	7.8 - 21.3
p-value^b^	< 0.01		0.05		0.11	
						
Marital status at interview						
Single/not cohabiting	6.8%	3.0 - 14.8	16.7%	9.7 - 27.1	28.4%	18.8 - 40.5
Married/long-term relationship	4.1%	2.6 - 6.6	11.6%	8.4 - 15.7	14.6%	10.8 - 19.5
Divorced/separated/widowed	5.8%	1.9 - 16.3	19.8%	10.8 - 33.4	23.3%	13.4 - 37.4
p-value^b^	0.56		0.20		0.02	
						
Vulnerability factors						
0 or 1	5.5%	2.4 - 12.1	6.0%	2.9 - 11.7	10.9%	5.6 - 19.9
2 or 3	2.0%	1.2 - 3.1	10.2%	6.0 - 16.7	13.7%	8.9 - 20.6
4 or 5	2.4%	1.3 - 4.3	14.9%	8.5 - 24.8	25.8%	16.7 - 37.7
6+	10.3%	5.3 - 18.9	20.9%	13.8 - 30.5	26.5%	17.9 - 37.3
p-value^b^	< 0.01		0.01		0.01	
						
Rank^c^						
Officer	4.6%	2.0 - 10.4	3.6%	1.5 - 8.1	10.2%	5.9 - 17.0
Other rank	4.9%	3.2 - 7.5	17.5%	13.6 - 22.3	21.3%	16.7 - 26.7
p-value^b^	0.90		< 0.01		0.01	
						
Medical downgrading since Jan 2003						
No	4.9%	3.1 - 7.5	13.9%	10.5 - 18.2	20.0%	15.7 - 25.0
Yes	4.6%	2.0 - 10.3	12.0%	7.5 - 18.7	10.9%	6.4 - 18.0
p-value^b^	0.89		0.59		0.03	
						
Serving status at interview						
Serving	4.8%	3.0 - 7.6	12.0%	8.9 - 15.9	17.9%	13.9 - 22.8
Veteran	4.8%	2.7 - 8.5	19.0%	12.3 - 28.4	18.5%	11.5 - 28.4
p-value^b^	> 0.99		0.08		0.91	
						
Service						
Naval service	2.7%	1.3 - 5.6	5.2%	2.9 - 9.2	9.2%	3.8 - 20.8
Army	6.2%	4.0 - 9.5	13.0%	9.7 - 17.1	21.6%	16.9 - 27.1
Royal Air Force	1.2%	0.6 - 2.6	20.3%	12.1 - 32.0	11.1%	5.9 - 20.0
p-value^b^	< 0.01		0.02		0.02	
						
Role in parent unit at phase 1						
Combat	7.9%	4.2 - 14.5	14.5%	8.9 - 22.8	26.4%	17.5 - 37.7
Combat support	2.2%	1.0 - 4.8	9.9%	3.8 - 23.4	26.8%	13.5 - 46.2
Combat service support	4.4%	2.6 - 7.3	13.4%	9.9 - 17.9	13.2%	9.8 - 17.6
p-value^b^	0.10		0.76		0.01	
						
Status at phase 1						
Regular	5.1%	2.8 - 9.1	15.5%	10.9 - 21.6	21.4%	15.6 - 28.2
Reserve	4.5%	2.8 - 7.1	11.3%	8.2 - 15.4	14.4%	10.4 - 19.5
p-value^b^	0.74		0.18		0.07	
						
Previous deployments (by status at phase 1)						
Regulars & reserves:						
None	1.5%	0.8 - 2.9	9.7%	5.7 - 16.0	14.2%	8.5 - 22.9
TELIC 1	5.8%	3.4 - 9.8	13.1%	8.8 - 19.0	16.9%	11.5 - 24.1
TELIC 2 or later	4.3%	2.1 - 8.5	17.2%	11.2 - 25.6	25.2%	17.8 - 34.4
Other recent deployments	10.2%	3.5 - 26.2	13.7%	5.6 - 29.6	12.1%	4.8 - 27.1
p-value^b^	0.06		0.46		0.14	
						
Regulars:						
None	2.4%	1.0 - 5.6	10.5%	4.4 - 23.0	16.6%	7.1 - 34.1
TELIC 1	4.8%	1.6 - 13.9	12.8%	6.2 - 24.6	23.5%	13.4 - 37.9
TELIC 2 or later	4.6%	1.8 - 11.2	20.2%	12.1 - 31.9	26.4%	17.0 - 38.5
Other recent deployments	9.5%	2.5 - 29.6	15.0%	5.5 - 35.0	11.8%	4.0 - 30.0
p-value^b^	0.46		0.55		0.40	
						
Reserves:						
None	1.0%	0.4 - 2.7	9.2%	4.7 - 17.4	12.7%	6.6 - 23.1
TELIC 1	6.5%	3.6 - 11.4	13.3%	8.4 - 20.4	12.3%	7.3 - 19.9
TELIC 2 or later	3.4%	1.6 - 7.0	10.7%	5.5 - 19.8	22.6%	12.7 - 36.9
Other recent deployments	13.3%	2.3 - 49.7	8.3%	2.9 - 21.5	13.3%	2.3 - 49.7
p-value^b^	0.04		0.66		0.43	

^a ^Weighted to take account of sample weights

^b ^For chi-squared test comparing prevalence across the categories of the independent variable

^c ^Rank at interview for serving personnel; last held rank for veterans

^d ^Any depressive syndrome, any anxiety syndrome or somatisation disorder

**Table 3:** Associations of PTSD symptoms with status and deployment history, odds ratio (OR)^a ^and 95% confidence intervals (CI)

	**PTSD symptoms**	
	
**Variable**	**Unadjusted OR (95% CI)**	**Adjusted OR^c ^(95% CI)**
Status at phase 1		
Regular^b^	-	-
Reserve	0.87 (0.40-1.92)	0.80 (0.31-2.06)
		
Previous deployments (by status at phase 1)		
Regulars & reserves:		
None^b^	-	-
TELIC 1	3.95 (1.65-9.44)	3.43 (1.20-9.79)
TELIC 2 or later	2.86 (1.06-7.69)	1.81 (0.61-5.40)
Other recent deployments	7.34 (1.97-27.3)	6.64 (1.60-27.6)
		
Regulars:		
None^b^	-	-
TELIC 1	2.10 (0.49-9.05)	1.09 (0.23-5.20)
TELIC 2 or later	2.01 (0.54-7.44)	0.55 (0.17-1.79)
Other recent deployments	4.31 (0.83-22.5)	1.73 (0.34-8.71)
		
Reserves:		
None^b^	-	-
TELIC 1	6.85 (2.09-22.5)	6.88 (1.86-25.4)
TELIC 2 or later	3.48 (0.98-12.4)	1.88 (0.42-8.33)
Other recent deployments	15.3 (1.83-127.3)	21.9 (2.67-178.9)

^a ^Odds ratios relate to the odds of having the diagnosis, weighted to take account of sample weights

^b ^Reference group for odds ratio

^c ^Adjusted for status, previous deployments, educational attainment, vulnerability factors and Service

PTSD symptoms were associated with being male, lower educational attainment, having greater pre-enlistment vulnerability and being in the Army. Neurotic disorders were associated with lower educational attainment, greater pre-enlistment vulnerability, holding a lower rank, and being in the Royal Air Force. Alcohol abuse was associated with younger age, being male, not being in a relationship, greater pre-enlistment vulnerability, holding a lower rank, being in the Army, having a combat or combat support role and not being medically downgraded.

#### Previous deployments

We present the deployment status analysis by engagement type due to our previous finding of a health effect among reserves [9]. The unadjusted PTSD prevalence was associated with deployment when regulars and reserves were examined together due to an increase in prevalence in those who deployed on TELIC 1, and other recent deployments (predominately deployment to Afghanistan). Analysis by engagement type shows that this effect is restricted to reserves only. There was no effect of deployment on the prevalence of neurotic disorders or alcohol abuse.

The association between PTSD symptoms and deployment persists for TELIC 1 and other recent deployments when regulars and reserves are examined together after adjustment. Repeating this analysis by engagement type, the deployment effect was observed for reserves only. There was no effect of deployment on the prevalence of neurotic disorders or alcohol abuse.

### UK versus US comparisons (Table 4)

**Table 4:** Combat experiences and mental heath for Army personnel post deployment to Iraq by regular/reserve status, UK vs. US data^a^, mean or percentage^b^, with 95% confidence interval^c^

	**UK data**		**US data**	
	
	**Regulars**	**Reserves**	**Active duty**	**National Guard & Reserves**
Age (years)^d^	36.9 (35.7-38.0)		30.4 (30.3-30.5)	
Male^d^	90.4 (85.6-93.7)		90.8 (90.6-91.0)	
Married^d^	60.0 (52.4-67.1)		58.2 (57.9-58.5)	
				
Combat experiences^e^				
Witnessed someone wounded or killed	55.9 (44.5-66.7)	42.5 (33.5-52.1)	53.6 (53.2-54.0)	53.9 (53.4-54.5)
Discharged weapon	20.8 (12.9-31.9)	10.8 (6.0-18.8)	25.2 (24.9-25.6)	24.1 (23.6-24.6)
Felt in danger of being killed	68.0 (55.8-78.2)	68.1 (57.7-76.9)	49.0 (48.6-49.4)	55.3 (54.8-55.9)
1 or more	76.2 (65.2-84.6)	71.5 (61.6-79.7)	66.5 (66.1-66.9)	69.6 (69.1-70.1)
				
PHQ-2 depression screen, number of positive responses				
1	12.5 (6.7-22.3)	7.9 (4.5-13.6)	6.2 (6.0-6.4)	7.3 (7.1-7.6)
2	4.3 (1.9-9.7)	4.4 (1.7-11.0)	4.2 (4.0-4.3)	5.6 (5.4-5.9)
1 or more	16.8 (10.1-26.7)	12.3 (7.5-19.5)	10.3 (10.1-10.6)	13.0 (12.6-13.3)
				
Primary care - PTSD screen^f^, number of positive responses				
1	15.9 (9.2-25.9)	19.1 (12.6-28.0)	12.3 (12.0-12.6)	14.8 (14.4-15.2)
2	8.0 (3.9-15.9)	2.8 (1.7-4.6)	7.7 (7.4-7.9)	10.2 (9.9-10.5)
3	3.5 (1.3-9.1)	3.0 (1.8-4.9)	4.9 (4.8-5.1)	7.0 (6.7-7.3)
4	2.5(0.7-9.1)	4.0 (1.7-9.4)	4.1 (4.0-4.3)	7.3 (7.0-7.6)
2 or more	14.1 (8.3-22.9)	9.7 (6.4-14.7)	16.7 (16.4-17.0)	24.5 (24.0-25.0)
3 or more	6.1 (2.7-12.9)	7.0 (4.0-11.8)	9.1 (8.8-9.3)	14.3 (14.0-14.7)
				
Suicidal ideation	0.5 (0.2-1.6)	1.4 (0.3-7.5)	0.6 (0.5-0.7)	1.5 (1.3-1.6)
				
Fair or poor overall health assessment	14.0 (8.5-22.2)	11.0 (7.5-16.1)	16.5 (16.2-16.8)	20.8 (20.3-21.2)

^a ^US data from Milliken et al (2007)

^b ^Mean age; percentage for all other variables

^c ^UK data weighted to take account of sampling weights

^d ^Demographic data are not separately available for those on active duty and those who are National Guard or reserves; data are shown are for US or UK personnel overall

^e ^Data on combat experiences colleted at phase 1 for the UK study (i.e. combat experiences data collected before health outcome measures). Combat and health outcome data from the US study collected at the same time

^f ^In the UK study, the PC-PTSD screen was only asked for those individuals who endorsed a criterion A event

We compared our data with the US Post Deployment Health Reassessment Study (PDHRA) [20], specifically Army personnel who had served in Iraq. The UK sample was older than the US sample but the two samples were similar with regards to gender and marital status. Deployment experiences of regular forces were broadly similar, except that UK personnel were more likely to report that they felt in danger of being killed. US reserve forces reported witnessing someone wounded or killed and discharging their weapon significantly more than UK reserves, while feeling in danger of being killed was more frequently reported among UK reserves.

The rates of depressive disorder and suicidal ideation were comparable between the US and UK for both regulars and reserves. Rates of PTSD symptoms were not significantly different amongst regulars but they were significantly higher for US military reserves than UK reserves. However, this difference disappears when the samples are further stratified by whether or not reservists discharged their weapon in combat (data available from authors). Fair or poor assessment of health based on the SF-36 were comparable for UK and US regulars, but significantly more frequently reported in US reserves than their UK reserve counterparts.

## Discussion

Mental disorders are common in the UK military, especially alcohol problems and neurotic disorders. PTSD remains relatively uncommon. There is no health effect of deploying during the 2003 invasion of Iraq (TELIC 1) for regular personnel, but reservists who deployed on TELIC 1 and other recent non-TELIC deployments are at an increased risk of PTSD symptoms compared to reservists who do not deploy.

### Common diagnoses

The high prevalence of alcohol problems is consistent with our previous reports [24]. Depression is also common (as it is in general population studies and the US studies reported below) although major depressive disorder is less common than milder depressive disorders. Panic disorder is rare, presumably individuals who suffer from severe panic symptoms would have difficulty in completing routine operational duties, pre-deployment training, or pre-enlistment screening. Somatisation disorder was uncommon which is consistent with the lack of increased prevalence of medically unexplained symptoms associated with deployment to Iraq in contrast to the 1991 Gulf War [29]. However, recent data shows that reporting of (all) symptoms has increased since the 1991 Gulf War (Horn O, Sloggett A, Ploubidis GB, Hull L, Hotopf M, Wessely S, Rona RJ: Upward trends in symptom reporting in the UK Armed Forces submitted 2008).

### Associations of common mental health problems and PTSD symptoms

#### Socio-demographics

Young men, those with pre-enlistment vulnerability and those who have been separated, divorced or widowed were at increased risk of common mental disorders. It has already been well-documented that such groups are at greater risk of mental health problems within the military [25,26].

#### Military factors

For regular personnel, we did not find an overall health effect of deployment to the main war fighting phase of the Iraq War which contrasts with US findings [4,6]. However, in common with our previous study [9], we found a higher prevalence of PTSD symptoms in reserve personnel who deployed on TELIC 1 or other recent non-TELIC deployments when compared to non-deployed reservists. We have proposed that the increase in mental health problems in Iraq deployed reserves may be due to a higher perceived exposure to traumatic experiences in theatre, lower unit cohesion and morale amongst reservists, more marital discord during deployment and greater difficulties adjusting to life on homecoming [27].

#### Comparison with the general population

Direct comparison with a non-military population is not possible as the PHQ has not yet been used in large scale epidemiological surveys in the UK. The most comprehensive survey of the mental health of the UK general population occurred in 2000 utilizing the Clinical Interview Schedule - Revised (CIS-R) [28]. The prevalence of neurotic disorders (generalized anxiety, depression and panic) in the UK population is 16.4% compared to 13.0% in this military sample. We would expect the prevalence of neurotic disorders to be lower in the military because of the screening procedures prior to enlistment, and the discharge of the most unwell after recruitment. Prevalence estimates of depression were similar between the military (11.0%) and the general population (11.0%), as was panic disorder (military 1.1%, general population 0.7%), major depression (military 3.7%, general population 2.6%) and somatisation (military 1.8%, general population 2.6%).

### Comparison with other military populations

#### 1991 Gulf War Studies

After the 1991 Gulf War, a series of case-control studies, comparing the health of Gulf and non-Gulf deployed personnel were conducted [29-31], including detailed clinical psychiatric assessment [32]. In spite of methodological differences, amongst non-disabled Gulf veterans, Ismail et al [32] reported that the four week prevalence of major depressive disorder was 3% (compared with 3.7% in our study), the prevalence of panic disorder was 1% in comparison to 1.1% in our cohort, and the prevalence of any anxiety disorder was 3% in contrast to 4.7% in our cohort. The major difference was in relation to alcohol problems. Ismail et al [32] report a prevalence of 7% for alcohol dependence and 3% for alcohol problems, in contrast to a combined prevalence of 18.3% in this cohort. The difference may be due to changes in the culture of drinking in the UK in general and the Armed Forces in the last decade [33,34], although the measures used were different in the two studies. Prevalence of PTSD in this study is higher than those reported in 1995 but the increase in the UK has been modest. Finally, Ismail et al [32] reported prevalence of somatoform disorder of 18.0% in unwell Gulf veterans and 6.0% in well Gulf veterans in comparison to rates of 1.8% in this cohort. This is consistent with the lack of an increase in medically unexplained symptoms after the 2003 Iraq conflict, in contrast to the 1991 Gulf War [35], after which there was an unexplained increase of medically unexplained symptoms (Gulf War Syndrome).

#### Contemporary US Studies

Riddle et al have reported on the prevalence of common mental disorders in a large military cohort in the US (The Millennium cohort) [26]. In spite of the methodological differences in sampling and some of the instruments used prevalence between the UK and the US cohorts are similar. Alcohol abuse was the most common diagnosis in the two studies (12.6% in the US versus 18.0% in the UK). The prevalence of major depressive disorder and panic disorder were similar (3.2% (US) versus 3.7% (UK) and 1.0% (US) versus 1.1% (UK)). The prevalence of other anxiety disorders was lower in the US when compared to the UK (2.0% and 3.8% respectively), whereas the prevalence of PTSD in our UK sample was 4.8% and 2.4% in the Millennium cohort.

We have previously reported the high prevalence of problem drinking in the UK military [24]. It is possible that the differences in the prevalence of alcohol problems between the UK and US found here may reflect differences in the culture of drinking or differences in the rate of deployment in the two samples as alcohol misuse increases after deployment [36].

There were no significant differences in the prevalence of PTSD symptoms between the US and UK regular personnel within similar demographic and deployment groups in this study. US reserve forces reported more PTSD symptoms than their UK counterparts, but this difference became non-significant when combat experience was taken into account. It is unclear why UK reserves felt more at risk of being killed or injured than their US counterparts despite their lower combat exposure, but this may be explained by differences in training and experience between US and UK reserves.

Initial comparisons between US and UK prevalence of PTSD after the Iraq War revealed differences using an identical measure of PTSD [9]. The current analysis supports Hoge and Castro's [37] suggestion that these differences are probably best explained by differences in demographics, military and combat experiences in the original study populations used for comparison.

### Strengths and limitations

The strengths of this study are the relatively large sample and high response rate, with no evidence of bias in terms of health between responders and non-responders. The study used a structured diagnostic instrument, and did not rely on questionnaire self-report of symptoms or distress. However, this is a cross-sectional study thus causal relationships cannot be inferred.

Although our response rate was high, our sample was already based on a 61% response rate [9]. It is possible that the lack of difference between responders and non-responders in both studies missed the most vulnerable, unwell or socially excluded members of the still serving/ex-military population.

For some of our subgroups, we had small numbers which inevitably has reduced the precision of our prevalence estimates. In contrast to other work, we reported lower rates of mental health disorder in female personnel. We are concerned that our results for women are likely to be distorted by the low numbers of women in the sample.

The comparisons that we make with US data are limited in several ways. First, although the data relate to the same Iraq deployment, they were collected in different ways. The PDHRA data was collected cross-sectionally in 2005-6 and enquiry was made about both exposure and PTSD symptoms at the same time. The UK data were collected at two time points (exposure was enquired about in the Phase 1 KCMHR military health study (2005-6)) and the PTSD data were collected 18 months-2 years later in the clinical interview study described in this paper. Second, the measure of PTSD used in the UK required endorsement of a Criterion A event in order to make the diagnosis, but this was not the case in the US study. Finally, our PTSD diagnoses were based on telephone interview rather than questionnaire report.

Although the PHQ is a well used measure, like all screens for mental disorders, it has limitations. Many argue that the existing measures in use for common disorders and PTSD are simply unable to sift out those with symptoms which result in functional impairment, and constitute disorders [38-40].

Although the study was independent of the military and results were entirely confidential, Service personnel may have been reticent to admit to mental health problems leading to an underestimation of true prevalence. This is particularly true of symptoms of 'alcohol abuse', the diagnosis of which may have disciplinary consequences for still serving personnel.

## Conclusion

There are three implications of this work. The first is that PTSD symptoms are not the main source of psychiatric morbidity in Service personnel. Alcohol misuse and depressive disorders are much more common and therefore should be the primary focus for education/prevention and intervention. Second, this study suggests that reservists remain at special risk of operational stress injury and this risk extends beyond those who served in the initial war fighting period of the Iraq war. Thus initiatives in the UK to provide enhanced assistance to reservists are still pertinent. Finally, we have not replicated the previous reports of substantial differences in the prevalence of post traumatic stress disorder symptoms between US and UK troops deployed to Iraq.

## Competing interests

This study was funded by UK's Ministry of Defence contract number R&T/1/0078. The authors' work was independent of the funders, and we disclosed the paper to the Ministry of Defence at the point we submitted it for publication.

NG is a full-time active service medical officer who has been seconded to the Academic Centre for Defence Mental Health, SW is Honorary Civilian Consultant Advisor in Psychiatry to the British Army and JHH is a civilian employee of the Ministry of Defence. MH and SW are partially funded by the South London and Maudsley NHS Foundation Trust/Institute of Psychiatry National Institute of Health Research Biomedical Research Centre. All other authors declare that they have no conflicts of interest. The study received approval from the UK's Ministry of Defence (Navy) personnel research ethics committee and the King's College Hospital local research ethics committee.

## Authors' contributions

AI designed the study in collaboration with fellow authors, conducted a proportion of the interviews, assisted with the planning of the analysis, and prepared the first draft of this article for submission. LVS assisted in the design of the study, conducted a proportion of the interviews, and contributed to the article submitted. TB and JHH conducted a proportion of the interviews and contributed to the article submitted. JH assisted in the design of the study. NG and LH contributed to the article submitted. RR assisted with the planning of the analysis and contributed to the article submitted. MH assisted with the planning of the analysis and contributed to the article submitted. SW contributed to the design of the study, and contributed to the article submitted. NF assisted in the design of the study, led on the planning and execution of the analysis and co-wrote the article submitted. All authors read and approved the final manuscript.

## Pre-publication history

The pre-publication history for this paper can be accessed here:



## Supplementary Material

Additional file 1 **Supplementary table 1 (S1) and Supplementary table 2 (S2)**. Table S1: Sampling weights used to generate weighted prevalences, number and percentage (%) within each 2 × 2 table: The data provided presents the weighted prevalences. Weighting was based on the inverse of the sampling weight for the three characteristics that were over-sampled in the study compared to the cohort sample. Table S2: Supplementary Table S2: Characteristics of responders and non-responders in the KCMHR clinical cohort, number (%), odds ratio^a^, and 95% confidence interval (CI): The data provide a comparison of responders and non-responders. Click here for file
