# Supplementary material for: The prevalence of common mental disorders and PTSD in the UK military: using data from a clinical interview-based study
Source: BMC Psychiatry. 2009 Oct 30;9:68. doi: 10.1186/1471-244X-9-68 (PMC2774683; doi:10.1186/1471-244X-9-68)
Supplement: Additional file 1 — Supplementary table 1 (S1) and Supplementary table 2 (S2). Table S1: Sampling weights used to generate weighted prevalences, number and percentage (%) within each 2 × 2 table: The data provided presents the weighted prevalences. Weighting was based on the inverse of the sampling weight for the three characteristics that were over-sampled in the study compared to the cohort sample. Table S2: Supplementary Table S2: Characteristics of responders and non-responders in the KCMHR clinical cohort, number (%), odds ratioa, and 95% confidence interval (CI): The data provide a comparison of responders and non-responders. [file 1471-244X-9-68-S1.docx]

Supplementary Table S1: Sampling weights used to generate weighted prevalences, number and percentage (%) within each 2 x 2 table

|  |  | KCMHR military health study (phase 1), N=10,036^a^ | | | | |  | KCMHR clinical cohort, n=821 | | | | |  | Sampling weights | | |
| --- | --- | --- | --- | --- | --- | --- | --- | --- | --- | --- | --- | --- | --- | --- | --- | --- |
|  |  | GHQ non-case | |  | GHQ case | |  | GHQ non-case | |  | GHQ case | |  | GHQ non-case |  | GHQ case |
|  |  | N | (%) |  | N | (%) |  | n | (%) |  | n | (%) |  |  |  |  |
| Regulars |  |  |  |  |  |  |  |  |  |  |  |  |  |  |  |  |
| PCL non-case |  | 6,772 | (79.8) |  | 1,394 | (16.4) |  | 107 | (25.1) |  | 133 | (31.1) |  | 3.2 |  | 0.5 |
| PCL case |  | 44 | (0.5) |  | 281 | (3.3) |  | 18 | (4.2) |  | 169 | (39.6) |  | 0.1 |  | 0.08 |
|  |  |  |  |  |  |  |  |  |  |  |  |  |  |  |  |  |
| Reserves |  |  |  |  |  |  |  |  |  |  |  |  |  |  |  |  |
| PCL non-case |  | 1,213 | (78.5) |  | 264 | (17.1) |  | 146 | (37.1) |  | 194 | (49.2) |  | 2.1 |  | 0.3 |
| PCL case |  | 6 | (0.4) |  | 62 | (4.0) |  | 4 | (1.0) |  | 50 | (12.7) |  | 0.4 |  | 0.3 |

^a^ Excludes 195 regulars and 41 reservists with missing data on GHQ caseness or PCL caseness

Supplementary Table S2: Characteristics of responders and non-responders in the KCMHR clinical cohort, number (%), odds ratio^a^, and 95% confidence interval (CI)

|  | Responders, n=821 | | Non-responders, n=262 | Odds ratio (95% CI) | |
| --- | --- | --- | --- | --- | --- |
| Variable | n | (%) | n (%) | Unadjusted | Adjusted^c^ |
| Age at phase 1 (years) | |  |  |  |  |
| <25 | 90 | (11.0) | 60 (23.0) | 2.99 (1.96-4.57) | 2.19 (1.30-3.69) |
| 25-29 | 116 | (14.1) | 52 (19.9) | 2.01 (1.32-3.08) | 1.61 (1.01-2.57) |
| 30-34 | 163 | (19.9) | 50 (19.2) | 1.38 (0.91-2.09) | 1.18 (0.77-1.83) |
| 35-39 | 160 | (19.5) | 34 (13.0) | 0.95 (0.60-1.51) | 0.85 (0.53-1.36) |
| 40+^b^ | 292 | (35.6) | 65 (24.9) | - | - |
|  |  |  |  |  |  |
| Sex |  |  |  |  |  |
| Male^b^ | 720 | (87.7) | 228 (87.4) | - | - |
| Female | 101 | (12.3) | 33 (12.6) | 1.03 (0.68-1.57) | - |
|  |  |  |  |  |  |
| Rank at phase 1 | |  |  |  |  |
| Officer | 168 | (26.0) | 31 (13.7) | 0.60 (0.39-0.91) | 0.69 (0.45-1.07) |
| Non-commissioned officer^b^ | 510 | (78.8) | 157 (69.2) | - | - |
| Other rank | 137 | (21.2) | 70 (30.8) | 1.66 (1.18-2.33) | 1.19 (0.81-1.76) |
|  |  |  |  |  |  |
| Service |  |  |  |  |  |
| Naval service | 101 | (14.0) | 36 (16.0) | 1.15 (0.76-1.73) | - |
| Army^b^ | 588 | (81.7) | 183 (81.3) | - | - |
| Royal Air Force | 132 | (18.3) | 42 (18.7) | 1.02 (0.70-1.50) | - |
|  |  |  |  |  |  |
| Status at phase 1 | |  |  |  |  |
| Regular^b^ | 427 | (52.0) | 166 (63.6) | - | - |
| Reserve | 394 | (48.0) | 95 (36.4) | 0.62 (0.47-0.83) | 0.79 (0.57-1.10) |
|  |  |  |  |  |  |
| Serving status at phase 1 | |  |  |  |  |
| Serving^b^ | 701 | (85.7) | 216 (83.4) | - | - |
| Veteran | 117 | (14.3) | 43 (16.6) | 1.19 (0.81-1.75) | - |
|  |  |  |  |  |  |
| Cohort at phase 1 | |  |  |  |  |
| ERA^b^ | 382 | (46.5) | 125 (47.9) | - | - |
| TELIC 1 | 439 | (53.5) | 136 (52.1) | 0.95 (0.72-1.25) | - |
|  |  |  |  |  |  |
| Ethnic group |  |  |  |  |  |
| White^b^ | 665 | (97.5) | 211 (95.5) | - | - |
| Non-white | 17 | (2.5) | 10 (4.5) | 1.85 (0.84-4.11) | - |
|  |  |  |  |  |  |
| PCL case at phase 1 | |  |  |  |  |
| No^b^ | 580 | (70.6) | 171 (65.5) | - | - |
| Yes | 241 | (29.4) | 90 (34.5) | 1.27 (0.94-1.70) | - |
|  |  |  |  |  |  |
| GHQ case at phase 1 | |  |  |  |  |
| No^b^ | 275 | (33.5) | 80 (30.7) | - | - |
| Yes | 546 | (66.5) | 181 (69.3) | 1.14 (0.84-1.54) | - |
|  |  |  |  |  |  |
| Fitness to deploy at phase 1 | |  |  |  |  |
| Unfit^b^ | 60 | (9.5) | 27 (12.7) | - | - |
| Fit | 574 | (90.5) | 185 (87.3) | 0.72 (0.44-1.16) | - |

^a^ Odds ratios relate to the odds of being a non-responder

^b^ Reference group for odds ratio

^c^ Adjusted for age, rank and status
